# Loan repayment performance of micro and small-scale enterprise: evidence from North Wollo Zone, Ethiopia

**DOI:** 10.1016/j.heliyon.2022.e12085

**Published:** 2022-12-05

**Authors:** Ebrahim Endris

**Affiliations:** Department of Agricultural Economics, Woldia University, Woldia, Ethiopia

**Keywords:** Collateral, Default, Financial literacy, Loan repayment

## Abstract

Loan defaulting is a prominent challenge to financial institutions' outreach and sustainability. As it has been increasing and become a critical problem in the study area, this study aimed to analyze the loan repayment performance of Micro and Small-scale Enterprise (MSEs) in North Wollo Zone, Amhara Region, Ethiopia. The study used a multi-stage sampling technique to select 336 sample enterprises (181 non-defaulter and 155 defaulters). Both qualitative and quantitative data were collected from primary and secondary data sources. The descriptive statistics result showed that non-defaulting was higher in trade (29.28%) and manufacturing (21.55%) MSEs, while high defaulting pertains to MSEs engaged in agriculture (24.52%) and construction (21.93%) sectors, respectively. The findings of financial ratios revealed that the average current ratio, debt ratio, and debt-to-equity ratio were 0.65, 0.72 and 7.95, respectively. The logit regression result showed that enterprise manager education level, collateral security, and financial literacy positively and significantly affected loan repayment performance while distance to lending institutions, repayment period, and loan diversion negatively affected it. Therefore, this study recommends that lending institutions and other concerned organizations organize warranties for the loan, align repayment schedules with the production period of enterprises, inclusive monitoring and supervision, and provide financial and basic business training to enterprises.

## 1 Introduction

Financial inclusion helps to reduce income inequality in low-income and high-fragility countries specifically in a country in which the financial system is relatively weak (Kim, 2015). Sub-Saharan Africa region's financial inclusion still lagging and the gap remains significant (Sha'ban et al., 2019), which is very worrying and needs urgent attention (Ofori-Abebrese et al., 2020). Financial inclusion of the continent is hindered by lack of money resources, too expensive to use the services and lengthy distance to the nearest financial service provider (Ulwodi and Muriu, 2017). Financial inclusions of Micro, Small and Medium Enterprise (MSMEs) are vital as it has a significant contribution to national economic growth and development. In this regard, the sector doesn't reached its milestone and is confronted with multiple challenges. For example, 41% of developing countries formal MSMEs didn't obtained the needed finance, and this financial shortage is expected to be about $5 trillion, which is 1.3 times the existing lending to MSME (Moritán, 2020). The lack of financial access inhibited the startup and performance of Micro and Small-scale Enterprises (MSEs). Consequently, financially constrained firms in Sub-Saharan Africa have a 6.6% lower marginal revenue product of capital and are 15% less efficient than unconstrained firms (Amos and Zanhouo, 2019). The operational self-sufficiency of Ethiopian Micro-Finance Institutions (MFIs) is highest compared to all the regional averages (Wassie et al., 2019). The accessibility and financial sustainability of saving and credit cooperatives have a vital contribution to alleviating credit market failure by providing financial services to poor and low-income earning households (Henock, 2019).

The repayment behaviours can be influenced by many factors associated with consumer financial situations (e.g., consumer spending). Additionally, a substantial proportion of consumers who make contradictory choices and/or delay repayment to the maximum permissible time are very likely to be financially constrained (Li et al., 2022). In addition, moral hazard problems, lack of proper monitoring, high lending interest rate, inadequate collateral and nepotism have a significant positive impact on the raising of non-performing loans (Ghosh et al., 2020). Constraints in access to finance represent a significant barrier for youth entrepreneurs to start and scale up their businesses. Youth are perceived to be riskier clients because they tend to lack business experience, credit histories, savings and other assets to offer as collateral (WB, 2020).

There are different sources of finance for the startup of MSEs in Ethiopia. The primary sources of MSEs' initial capital are microfinance institutions, followed by banks and their own capital (Alemu, 2018). Even though the government has made substantial development in improving MSEs access to finance like youth revolving fund (FDRE, 2016, 2017), a high rate of matching funds and liquidity requirements constrained MSE's financial access from existing MFIs (Sissay, 2016). In addition, lack of collateral, complex loan procedures, high-interest rate, and difficulty to form groups constrained MSEs' access to finance (Lakew, 2018). There are client-related factors that impede financial institution performance and leading to a strict and complex lending system in Ethiopia. These include loan defaulting, loan diversion into non-income-generating purposes, business situations, and poor follow-up and monitoring (Alemayehu and Lemma, 2014; Dangisso and Deyganto, 2020). As a result of loan defaulting behaviour of MSEs, financial institutions became reluctant to give credit to young enterprises (Nega and Hussein, 2016). The loan repayment performance of MSEs is affected by the inconvenience of loan payback period, lack of financial skill and planning (Alemu, 2018).

Amhara Credit and Saving Institution (ACSI) is the largest MFI in Amhara Regional State of Ethiopia that provides financial services to marginalized small business operators and poor smallholder farmers (Chirkos, 2014). It has a significant contribution to women's economic empowerment, such as increasing women's role in resource controlling and asset possession, improve household income, and saving (Mandefro, 2010; Mengstie and Singh, 2019). Even though MFI has a significant contribution, there is an extremely insufficient loan and very restrictive bureaucracy and regulations to obtain the loan (Mandefro, 2010). Loan defaulting is a major challenge to financial institutions and startup enterprises, which hurt lending institution via incurring high loan collection cost, limiting credit outreach to new enterprise, and hindering financial sector development (Alemayehu and Lemma, 2014; Dangisso and Deyganto, 2020). The exsistence of poor loan repayment performance and increasing loan defaulting restricted the outreach and sustainability of financial institutions in the study area (NWZEDO, 2021). In 2020/21, 34% of the loan was not repaid (default), implying that MSEs do not repay their loan within the loan repayment maturity period (NWZ-ASCI, 2021). Even though loan defaulting is such critical problem in MSEs of the study area, the existing empirical studies focused on farmers’ loan repayment and there is no evidence focused on small business loan repayment performance. Hence, this study aimed to fill the existing gaps through investigating factors affecting loan repayment performance of MSEs in the North Wollo Zone. The findings of this study provide empirical evidence and policy directions to financial institutions, enterprises, MSEs lending and supporting organizations, researchers, and government policy makers in the development of MSEs through unlocking the financial challenges of the sector.

The general objective of this study is to analyze factors affecting loan repayment performance of MSEs in North Wollo Zone, Amhara regional state, Ethiopia. Specifically, this study aimed to:

1) Assess the financial performance of MSEs in North Wollo Zone, Amhara regional state, Ethiopia
2) Identify determinants of loan repayment performance of MSEs in the study area

The rest of this paper is organized as follows. First research methodology used for this study were presented. Secondly result and discussion, which includes descriptive and econometrics results were discussed. Finally, conclusion and recommendations were presented, respectively.

## 2 Research methodology

### 2.1 Description of study area

**Figure 1:**
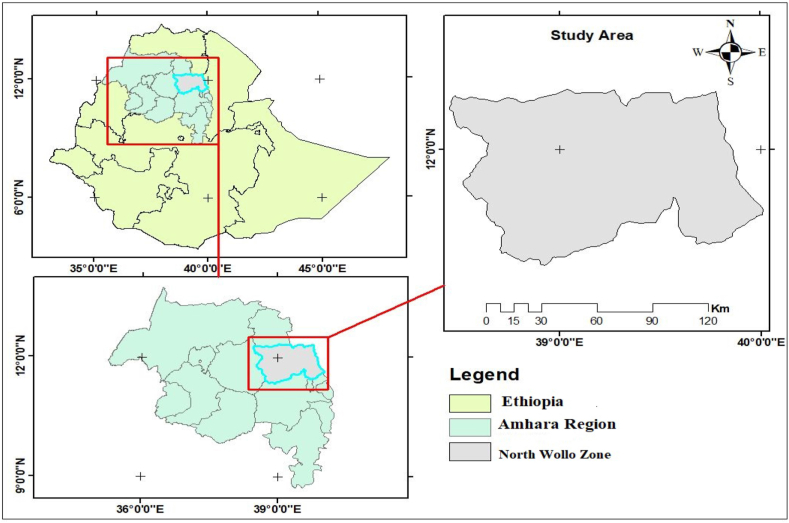
Study area map. **Source**: GIS Map (2022)

This study was conducted in the North Wollo Zone (Figure 1), which is part of the Amhara Regional State, Ethiopia. The zonal capital city (Woldia) is found at a distance of 521 km north of Addis Ababa, the country's capital city and 354 km from Bahir Dar, the regional city. The area coverage of the zone is 12, 172.5 km2 142,295.32 Ha (CSA, 2007) and is divided into 14 districts and 5 town administrations. The study area is enclosed by South Wollo zone on the south, South Gondar on the west, Wag-Hemra on the north, Tigray Region on the northeast, part of its southern border is defined by the Mille River. The overall population projection of North Wollo Zone in 2017 was 1, 824, 361 of which, 913,572 were male and 910, 789 were female. Of these population, 270,686 were urban residents and 1,553,674 were rural population (CSA, 2013). In 2018/19, about 9,112 MSEs were established and 21,958 permanent and temporary employments are created through micro and small-scale enterprise in the study area (NWZEDO, 2019).

### 2.2 Sampling procedure

The study used a multi-stage sampling procedure to select 336 MSEs in North Wollo Zone, Amhara regional state, Ethiopia. First of all, four districts were selected purposively from North Wollo zone based on the presence of large number of MSEs in the district. Secondly, loan beneficiary MSEs were selected from each district as only loan beneficiaries are targeted for this study. All borrower MSEs that repaid their loans by the due date are categorized as non-defaulters, whereas MSEs that did not repay their loan until 90 days after the due date are categorized as defaulters. Stratified sampling was applied in the third stage by categorizing defaulter and non-defaulter MSEs into two strata. Lastly, simple random sampling was applied in each category to obtain the intended sample enterprises.

This study adopted (Cochran, 1977) sampling formula (equation 1) to determine the sample size of the study. The Cochran sampling method was used as the population is heterogeneous in terms of loan repayment status (being defaulter and non-defaulter). The number of loan beneficiary MSEs in four chosen districts of North Wollo zone was 2690, of which 1241 are defaulters and 1449 are no-defaulters.

(1) $$n=\frac{Z^{2}pq}{e^{2}}$$

Where; Z-value for selected alpha level (95% degree of confidence)

$$n=\frac{{\left(1.96\right)}^{2}*0.5*0.5}{0.05^{2}}=384$$

The sample size is reduced to 336 using the following formula;

(2) $$n_{0}=\frac{n}{1+\frac{n-1}{N}}$$

Where ‘N’ (equation 2) is the total population (2690) and ‘n’ is the primary sample size (384). From this, 181 non-defaulter and 155 defaulters were selected proportionally (Table 1).

**Table 1:** Sample distribution across districts.

Districts	Total population		Non-defaulter		Defaulter	
	Population	Sample	Population	Sample	Population	Sample
Kobo district	695	87	371	46	324	41
Gubalafto district	1065	133	568	71	497	62
Woldia town	613	76	340	43	273	34
Meket district	317	40	170	21	147	18
**Total**	**2690**	**336**	**1449**	**181**	**1241**	**155**

### 2.3 Data sources and method of collection

The study used qualitative and quantitative data from both primary and secondary sources. The primary data was obtained from the sample MSEs (both defaulters and non-defaulters) using semi-structured interview. Pre-testing of the questionnaire was carried out, and a few amendments were made to the questionnaire. The questionnaire contains numerous questions about the socio-economic, and institutional characteristics of the sample enterprise which include the age of the enterprise, amount of loan obtained, suitability of loan repayment period, collateral security, distance to a lending institution, loan scheme etc. were collected through a face-to-face interview. In addition, focus group discussions and key informants interviews with loan officers, MSEs experts, and MFI branch managers were conducted to obtain supplementary data which was not obtained from enterprises interview. Secondary data was obtained from zone and district relevant offices (ACSI, micro and small enterprise office), different national and international reports, journals, and organizations.

### 2.4 Method of data analysis

Descriptive statistics and econometric model were used to investigate loan repayment performance of MSEs in the study area. Descriptive statistics such as percentage, frequency and t-test (chi-square) tests along with financial ratios of enterprises were used. The financial ratios such as current ratio, debt ratio, and debt to equity ratio were used to measure enterprise liquidity and solvency.

#### 2.4.1 Model specification

If responses are binary, the individual faces a pair of choices and makes that choice between the two alternatives (Greene, 2012) can be modelled in logit and probit functional forms. These two models are comparable except for the tails, and the logit resembles logistic distribution, whereas the probit has a standard normal distribution. Hence, these two models provide similar results except for the presence of numerous observations in the tails (Baltagi, 2011; Das, 2019).

The dependent variable takes a value of 0 or 1 subject to whether or not a borrower is a defaulter. Thus, the most widely used discrete choice model for qualitative response-dependent variables are the logit and probit models (Dougherty, 2001). Logit regression model estimates the probability of borrowers being defaulters or not, by predicting a binary dependent outcome from a set of explanatory variables. This study used logit model to analyze the association between explanatory variables and independent variables (loan repayment performance of MSEs). In this study, $Y_{i}$ is the dependent variable (loan repayment status) and $X_{i}$ is the independent variables. Denoting $Y_{i}$ = 1 if loan repaid on time and $Y_{i}$ = 0 if the enterprise had at least one missed/late repayment after 90 days of due date.

According to (Gujarati, 2004), the general logistic regression model showed as follows:

(3) $$Y=\ln\left(\frac{p}{1-p}\right)=\beta_{0}+\beta_{1}X_{1}+\beta_{2}X_{2}++\beta_{i}X_{i}+\varepsilon_{i}$$

The dependent variable Y has two responses, “1” treated as the MSEs are non-default in payment and “0” indicates MSEs are defaulters. ${X_{i}}^{\prime }s$ are explanatory variables, and $\beta_{i}$ are parameters to be estimated (equation 3). If p the probability that MSEs repay the loan on time, then 1−p is the proportion for defaulters and the odds of occurring of the first outcome is $\frac{p}{1-p}$.

Transformation of the odds $\frac{p}{1-p}$ using the natural logarithm gives the log odds or logit for of odds (equation 4). Then, the model is the log odds (equation 4) as a linear function of the explanatory variable as follows:

(4) $$E\left(y\right)=\frac{e^{\beta_{0}+\beta_{1}X_{1}+\beta_{2}X_{2}+.\;.\;.\;.\;.\;.\;.\;.+\beta_{i}X_{i}\;}}{1+e^{\beta_{0}+\beta_{1}X_{1}+\beta_{2}X_{2}+.\;.\;.\;.\;.\;.\;.\;.+\beta_{i}X_{i}\;}}$$

Where E(y) = p, the probability of success

**Variable Definition and Hypothesis**

The dependent variable is loan repayment performance of MSEs represented by a binary response of 1 if non-defaulter and 0 otherwise. The explanatory variables hypothesized to influence the loan repayment performance of MSEs are explained as follows:

**1. Enterprise age:** It is measured as the number of years of MSEs since its establishment. Enterprises, which stayed for many years, might have a stable business, and better experience in loan utilization and repayment. Therefore, enterprise age is hypothesized to affect the loan repayment performance of the enterprise positively.
**2. Manager's education:** It is the number of years of schooling attained by the enterprise manager or leader during the survey year. Education increase external business exposure improves the use of information, and business management skills, which enhances enterprise productivity and profitability. Therefore, the manger education level is hypothesized to foster the loan repayment performance of MSEs. The hypothesis is supported by (Kebede et al., 2016; Melese and Asfaw, 2019) who revealed that a higher education level increases loan repayment performance of the business.
**3. Loan size:** It is measured in natural logarithm of loan size obtained from the financial institution in Birr. The amount of loan might influence loan repayment performance of MSEs either positively or negatively. The higher loan size may increase borrowers’ burden to meet the large principal and interest obligations, while the increased loan enables enterprises to operate fully and productively which boosts loan repayment performance. The first hypothesis supported by (Melese and Asfaw, 2019; Salifu et al., 2018) that found large loan size became difficult for borrowers if businesses do not generate expected cash flows. The latter hypothesis conforms with (Enimu et al., 2017; Pasha and Negese, 2014) who revealed that sufficient loan size enables the business to operate at full capacity and increase loan repayment performance.
**4. Collateral security:** It is a dummy variable, which takes 1 if collateral for the loan is an asset, and 0 otherwise. The presence of reliable collateral is expected to increase the loan repayment performance of MSEs, as borrowers give attention to loan utilization and repayment to protect the loss of their collateral assets. This evidence is confirmed by (Abuye and Shiferaw, 2019) who found collateral is the main requirement to guarantee loan repayment practice of borrowers.
**5. Repayment period:** It is defined as the suitability of loan repayment period to the enterprise which helps them to arrange the required money to repay the debt. This is a categorical variable, which takes a value of 1 if the repayment period is quarterly, 2 if semi-annually, and 3 if annually. It is expected that a suitable repayment period for enterprises to have a positive effect. However, the loan repayment schedule, which does not consider business production and marketing period negatively affect the repayment rate. Hence, the shorter repayment period is expected to affect loan repayment performance negatively and vice versa. The hypothesis was confirmed by (Alemu, 2018) who revealed that loan payback period inconvenience negatively affects loan repayment of MSEs.
**6. Loan supervision frequency:** this variable is measured as the number of times per year loan supervision and monitoring has been done to enterprises. A continuous follow-up and supervision visit to evaluate loan utilization and repayment makes borrowers efficiently use their loan and remind their obligations. Therefore, it is expected to have a positive effect on the loan repayment performance of MSEs in the study area. The hypothesis is supplemented by (Agbeko et al., 2017) who found that intensive monitoring improves loan repayment rates. In addition, continuous follow-up and visit of respondents reduces the probability of being defaulter (Alemu, 2018; Enimu et al., 2017; Pasha and Negese, 2014).
**7. Loan utilization:** It is a dummy variable, which takes 1 if the loan is diverted for unintended purposes and 0 otherwise. The use of a loan for the unintended purpose whose productivity and profitability are not studied is expected to affect loan repayment performance negatively. This hypothesis is supported by (Kebede et al., 2016) who found that diverting loans into non-income-generating activities increases the default rate.
**8. Additional income source:** This is a dummy variable, which takes a value of 1 if the enterprise is involved in other income-generating activities and 0, otherwise. Such sources of income are expected to have a positive contribution toward loan repayment performance. Hence, enterprises involved in additional income-generating activities are expected to be more capable of repaying their loan in time. This evidence is supported by (Melese and Asfaw, 2019) who found that enterprises that have additional income sources positively affect loan repayment performance. This income may lead to an increase in loan repayment rates (Afolabi, 2017).
**9. Distance to lending institution:** This is the distance of enterprises' premises to credit-providing financial institutions in kilometres. Micro and small enterprise nearness to financing institutions lowers monitoring and transaction costs of the institutions, which increases loan repayment performance. Hence, distance to the lending institution is hypothesized to influence the loan repayment performance of the enterprise negatively. This is confirmed by (Jote, 2018) who revealed that borrowers’ nearness to lending institutions increases loan repayment.
**10. Financial literacy:** It is a continuous variable that is measured in the number of financial-related training provided for the enterprise members. Financial literacy helps to manage the financial records, which positively contributed to the loan repayment performance of enterprises. Therefore, financial literacy is expected to influence loan repayment positively. Well-organized training for borrowers minimizes the probability of being a defaulter (Jote, 2018; Pasha and Negese, 2014). Thus, financial literacy increases loan repayment significantly, which endorses the sustainability of financial institutions (Baidoo et al., 2020). On the other hand, lack of financial skills and planning negatively affects the loan repayment performance of MSEs (Alemu, 2018).
**11. Loan scheme:** It is a dummy variable that takes a value of 1 if loan is provided through an individual lending system and 0 if on a group basis. Commitment, trust, and satisfaction are all validated as characteristics of relationship quality between individual group members in the context of group lending (Tegambwage and Kasoga, 2022). For MSEs, collective financing helps banks to reduce the average approval and supervision costs and improves the financial efficiency of enterprises (Yuanyuan and Zongxian, 2019). Hence, this variable is expected to adversely affect the loan repayment performance of MSEs in the study area.

A summary of independent variables used for econometric model analysis was presented in Table 2 as follows;

**Table 2:** Definition of variables and hypothesis.

Independent variables		
Variable description	Definition	Expected effect
Enterprise age	Continuous: years of MSEs since the establishment	+
Manager's education	Continuous: years of schooling	+
Loan size	Continuous: the amount of loan received	**±**
Collateral security	Dummy: 1 for asset collateral; 0 for otherwise	+
Repayment period	Categorical: 1 if quarterly, 2 if half-annually (base), 3 if annually	-
Loan supervision	Continuous: frequency of loan supervision per year	+
Loan utilization	Dummy: 1 if loan diverted, and 0 otherwise	-
Additional income	Dummy: 1 Yes and 0, otherwise	+
Distance to MFI	Continuous: distance in kilometer	-
Financial literacy	Continuous: number of financial related training	+
Loan scheme	Dummy: 1 if individual lending and 0 if group lending.	-

## 3 Result and discussion

### 3.1 Descriptive results

The number of non-defaulter was higher in trade (29.28%) and manufacturing (21.55%) MSEs, and lower in service (17.68%), agriculture (17.13%) and construction (14.36%) sectors, respectively. Regarding default rate, high defaulting pertains to MSEs engaged in the agriculture (24.52%) and construction (21.93%) sectors, respectively (Table 3).

**Table 3:** Loan repayment performance of MSEs across different sectors.

Repayment status	MSEs sectors					
	Manufacturing	Construction	Service	Trade	Agriculture	Total
Non-defaulter	39	26	32	53	31	181
Defaulter	32	34	20	31	38	155
Total	71	60	52	84	69	336

#### 3.1.1 Loan and business characteristics (continuous variable)

The mean age of MSEs was 3.6 years and 41.37% of the enterprises are older than the mean age of MSEs in the study area. This indicates that the majority of MSEs were young and not well organized, which might hurt their financial performance, thereby loan repayment capacity. The average age of non-defaulter and defaulter MSEs was 3.67 and 3.52 years, respectively (Table 4). The t-statistics result showed that there is no significant relationship between the age of MSEs and the loan repayment performance of the enterprise. The mean level of enterprise manager education was 10 years of schooling with a minimum of 6 and a maximum of 17 years of schooling. This indicates that MSEs are established for youths to create employment for those school dropouts mainly above grade 10. There was a statistically significant difference between the mean education level of non-defaulter and defaulter enterprise managers at 1% significance level.

**Table 4:** Characteristics of MSEs enterprises.

Characteristics	Non-defaulter (181)			Defaulter (155)			Total (336)			t-value
	Mean	Minimum	Maximum	Mean	Minimum	Maximum	Mean	Minimum	Maximum	
Age of enterprise	3.67	2	8	3.52	2	8	3.60	2	8	0.88
education level	11.73	6	17	8.83	6	15	10.39	6	17	11.29∗∗∗
loan size	77513.48	5500	450000	70847.03	5000	370000	74438.18	5000	450000	0.77
distance to MFI	6.01	0.25	17	10.42	3	17	8.04	0.25	17	- 9.69∗∗∗
Financial literacy	2.46	1	4	1.34	0	3	1.94	0	4	12.99∗∗∗
Frequency of loan supervision	4.05	2	7	3.35	2	6	3.73	2	7	6.27∗∗∗

∗∗∗indicates statistically significance at 1%.

The average loan amount of MSEs in the study area was 74438 Birr, with a minimum of 5000 Birr and a maximum of 450000 Birr. There is no significant difference between the mean loan size of non-defaulter (77513.4) and defaulter (70847) enterprises. The mean distance of the enterprise working premise to the lending institution was 8 km with a minimum of 0.25 km and a maximum of 17 km. The average number of financial training for non-defaulter and defaulters was 2.46 and 1.34, respectively. In addition, loan supervision and monitoring on average were 4 times for non-defaulter and 3 times for defaulters annually. The t-test revealed that there is a significant difference between defaulters and non-defaulter in terms of the distance of the enterprise from lending MFI, financial literacy, and frequency of loan supervision at 1% significance level. This indicates that non-defaulter has less distance to lending MFI, obtained basic financial training, and frequent expert supervision than defaulters, which significantly enables them to have good loan repayment performance.

The t-statistics showed that there was a significant difference between non-defaulter and defaulters in distance to financial institutions, financial literacy, and loan supervision at 1% significance level.

The majority of the enterprise (56.84%) had taken less than 50,000 Birr, the other 22.62% took a loan between 50,001–100,000 Birr, and 20.54% received loan amount over 100,000 Birr (Table 5). In terms of repayment status, a large loan amount was received by non-defaulter than by defaulters. This might be due to lending institutions considering the socio-economic characteristics of the borrower when lending large loans.

**Table 5:** Loan size and repayment performance.

Category		Loan size (Birr)			Total
		<50,000	50,001–100,000	>100,000	
Non-defaulter	No	93	41	47	181
	%	51.38%	22.65%	25.97%	100%
Defaulter	No	92	27	36	155
	%	59.35%	17.42%	23.23%	100%
Total	No	185	68	83	336
	%	55.06%	20.24%	24.70%	100%

#### 3.1.2 Loan and business characteristics of MSEs (discrete variables)

The chi-square test showed that there was a significant difference between non-defaulter and defaulters in terms of collateral security at 1% significance level. The suitability of the repayment period was significant and the shorter period of repayment (quarterly) negatively influences loan repayment performance (Table 6). This is due to the shortage of time to produce business output and generate revenue to repay their debt. In addition, there was a significant difference between non-defaulter and defaulters in terms of loan utilization. Loan diversion to unintended purposes negatively and significantly affected loan repayment performance at 1% significance level. This might lead to the use of a loan for non-income-generating activities or projects whose viability is not studied increasing the probability of being defaulters. The presence of additional income sources increases loan repayment performance through increased income to repay the debt in time of MSEs do not generate sufficient revenue.

**Table 6:** Enterprise characteristics for dummy variable.

Variable		Non-defaulter (181)	Defaulter (155)	Total (336)	chi2
Collateral security	No	116	149	265	51.43∗∗∗
	Yes	65	6	71	
	Total	181	155	336	
Repayment period	Half-annually	99	44	143	-76.03∗∗∗
	Quarterly	61	24	85	
	Annually	21	87	108	
	Total	181	155	336	
Loan utilization	Diverted	11	57	68	-48.74∗∗∗
	Not-diverted	170	98	268	
	Total	181	155	336	
Additional income	No	116	115	231	3.97∗∗
	Yes	65	40	105	
	Total	181	155	336	
loan scheme	Group lending	114	96	210	0.039
	Individual lending	67	59	126	
	Total	181	155	336	

∗∗∗, and ∗∗ indicates statistically significance at 1%, and 5% level respectively.

The financial ratio of liquidity and solvency provides information about enterprises’ financial position to repay short-term and long-term loans. Table 7 shows the liquidity of enterprises by sector using the current ratio and enterprises' solvency to repay long-term loans depicted using the debt ratio and debt-to-equity ratio. The current ratio of enterprises is less than one (0.65) on average in the study area, which indicates MSEs have a liquidity problem to repay their current liabilities from their current asset. The current ratio was higher in trade, manufacturing, and agriculture enterprises respectively, while construction and service sector enterprises have a lower current ratio, which was difficult to repay their current debts.

**Table 7:** Summary of enterprises' financial ratios.

MSEs Sectors	Current Ratio			Debt Ratio			Debt to Equity Ratio		
	Mean	Min.	Max.	Mean	Min.	Max.	Mean	Min.	Max.
Manufacturing	0.84	0.41	1.23	0.87	0.68	0.98	8.25	7.44	9.32
Construction	0..68	0.42	0.96	0.79	0.72	0.91	8.42	6.95	9.23
Trade	0.87	0.63	1.18	0.58	0.47	0.86	7.43	5.82	8.56
Service	0.72	0.69	0.98	0.64	0.52	0.89	8.23	7.24	8.76
Agriculture	0.82	0.62	0.87	0.72	0.62	0.93	7.43	6.46	8.82
Total	0.65	0.554	1.044	0.72	0.602	0.914	7.952	6.782	8.938

**Source**: Survey (2021).

The debt ratio indicates whether the enterprise has high liability share from the enterprise's total assets or not. The average debt ratio for MSEs in the study area was 0.72, which indicates the enterprise has 72% of assets financed through loans. The mean debt ratios were larger in manufacturing and construction MSEs because enterprises in these sectors require a large investment, which is mainly financed through loans. The average debt-equity ratio was 7.95, which indicates enterprise debt in the study area was 7.95 times higher than the owner equity of the business. It helps to evaluate whether the enterprise asset is mainly financed by debt or owners’ equity. The higher ratio of debt financing raises the level of interest and principal repayment which increases the exposure of becoming a defaulter.

### 3.2 Econometrics results

The logit model output showed that the pseudo R2, Chi-square and p-value were 0.5706, 264.64 and 0.0000, respectively (Table 8). The logit regression model estimate showed that manager education level, collateral security, suitability of repayment period, loan utilization, distance to the lending institution, and financial literacy are significant variables influencing MSEs' loan repayment performance in the study area.

**Table 8:** Logit model result.

Variables	Odd ratio	St error	t-value	p-value
Enterprise age	1.095596	0.134	0.74	0.458
Manger education	1.603991	0.149	5.07∗∗∗	0.000
Loan size (ln)	0.802839	0.158	-1.12	0.264
Collateral security	6.028589	3.512	3.08∗∗∗	0.000
Repayment period (Semi-annually)				
Quarterly	0.2542596	0.125	-2.78 ∗∗∗	0.005
Annually	1.210734	0.543	0.43	0.670
Loan supervision	1.266304	0.261	1.14	0.252
Loan utilization	0.2594876	0.137	-2.55∗∗	0.011
Additional income	1.519201	0.674	0.94	0.646
Distance to MFI	0.8633235	0.038	-3.29∗∗∗	0.001
Financial literacy	3.002721	0.723	4.56∗∗∗	0.000
Loan scheme	1.390572	0.577	0.79	0.427
Constant	0.0148179	0.037	-1.66	0.098
Number of obs = 336		LR chi2(12) = 264.64		
Log likelihood = -99.570349		Prob > chi2 = 0.0000		
Pseudo R2 = 0.5706				

∗∗∗, and ∗∗ indicates statistically significance at 1%, and 5% level respectively.

#### 3.2.1 Enterprise manager education level

The result shows that manager education level had a positive relationship with loan repayment performance of MSEs and significantly at 1% significance level. An increase in manager education level by one year of schooling is associated with an increase in the odds of being a non-defaulter by a factor of 1.603, holding other factors constant. This is due to the fact that education enhances the skill and knowledge of enterprise managers which helps them to run their business in such a way it became sustainable, profitable, and repay their debt on time. This is consistent with the earlier hypothesis and consistent with the findings of (Kebede et al., 2016; Melese and Asfaw, 2019; Pasha and Negese, 2014), who noted that education improves borrower's ability to access and analyze business information, and helps to prepare the business plan and financial statement (Amene, 2017), which likely increases loan repayment performance. The finding is also consistent with the macroeconomic theory which states that human capital is the key determinant of non-defaulting on debt obligations (Inekwe, 2019). As education increases the loan repayment performance of enterprises, being illiterate increases the likelihood of becoming defaulters (Shahriar et al., 2019).

#### 3.2.2 Collateral security

The existence of collateral security positively and significantly affected MSEs loan repayment performance as hypothesized earlier with a 1% significance level. The presence of reliable loan collateral increases the likelihood of loan repayment because borrowers give high attention to loan repayment not to lose their collateral assets. The result implies that the probability of being non-defaulter increases by a factor of 6.023 for collateral-secured MSEs relative to others who don't have collateral for the loan. This is due to the fact that enterprises which have collateral already have the economic capacity and readiness to repay the loan. The finding is confirmed by (Abuye and Shiferaw, 2019) who found collateral assures loan repayment practice of clients.

#### 3.2.3 Repayment period

This variable negatively and significantly affects the loan repayment performance of MSEs at 1% significance level. The negative effect indicates that the presence of a short-period loan repayment schedule decreases the likelihood of being a non-defaulter significantly. The odds ratio for loan repayment period indicates that the probability of being non-defaulters decreased by a factor of 0.254 as the repayment period is scheduled quarterly relative to half-annually. The reality behind the finding is that the shorter repayment period was not suitable for enterprise production and marketing period, which became difficult to get cash for repaying their loans. This finding found consistent with (Alemu, 2018)) who founds that loan payback period inconvenience negatively affects MSEs' loan repayment. Hence, the arrangement of a suitable loan repayment period to sell their business output increases loan repayment performance (Kebede et al., 2016; Pasha and Negese, 2014).

#### 3.2.4 Loan utilization

Loan utilization for unintended action negatively and significantly influences borrowers’ loan repayment performance at 5% significance level. This negative relationship shows that the use of loans for unintended business decreases the probability of being non-defaulter by a factor of 0.259, keeping other factors constant. This might be due to the use of loans for non-targeted purposes whose productivity and profitability not assessed through a business plan may lead to business failure and poor loan repayment performance. The finding is consistent with (Kebede et al., 2016) findings who revealed that diverting loans into non-income-generating activities hurt loan repayment performance by increasing the default rate. However, the application of an entire loan for an intended and productive business lessens the probability of defaulting (Pasha and Negese, 2014).

#### 3.2.5 Distance to MFI

As it was hypothesized, distances of MSEs premise to the lending institution had a negative and significant influence on the loan repayment performance of MSEs. The logistic model result indicates that the likelihood of being non-defaulter decreases by a factor of 0.863, as the MSEs distance away from lending institutions increases by one kilometre. This might be because the long distance of MSEs from lending financial institutions reduces loan repayment performance due to difficulty in accessing lending institutions, limited access to information, and less institution follow-up. This finding supported with that an increase in transaction cost reduces enterprise loan repayment (Salifu et al., 2018). Likewise (Jote, 2018), found that borrowers’ nearness to institutions increases the possibility of borrowers repaying their loans.

#### 3.2.6 Financial literacy

It is measured in the number of financial-related training that found a positive and significant influence on MSEs loan repayment performance at 1% significance level. The odds ratio in favour of being non-defaulter increases by a factor of 3, as the number of financial training increases by one unit. The positive effect is due to the reality that financial literacy increases MSEs' skill and attitude to keep financial records and manage financial statements, which enhances the loan repayment performance of the enterprise. Training in business management, saving cultures, and credit management increase the possibility of being creditworthy borrowers (Jote, 2018; Melese and Asfaw, 2019). Enhancing financial literacy improves successful loan repayment (Baidoo et al., 2020). Conversely, lack of financial skill and planning negatively affects loan repayment of MSEs (Alemu, 2018). The finding links with (Pasha and Negese, 2014) who found that delivery of critical training properly for borrowers lessens the likelihood of being a defaulter.

## 4 Conclusion

Financial inclusions of MSEs are crucial since the sector has a significant role in national development. However, the high loan default rate has been increasing, which restricted the outreach and sustainability of financial institutions. Hence, this study aimed to examine factors affecting loan repayment performance of MSEs in North Wollo Zone, Amhara regional state, Ethiopia. Multi-stage sampling technique was adopted to select 336 sample MSEs (181 non-defaulter and 155 defaulters) in the study area. Primary and secondary data sources were used for collecting qualitative and quantitative data from sample enterprises. Both descriptive statistics and an econometric model were used to investigate the loan repayment performance of MSEs in the study area. Descriptive statistics such as percentage, frequency and t-test (chi-square) tests, including financial ratios of enterprises were used. Logit model was used to identify factors affecting loan repayment performance of MSEs. The number of non-defaulter was higher in trade (29.28%) and manufacturing (21.55%) MSEs, and high default pertains in MSEs engaged in agriculture (24.52%) and construction (21.93%) sectors, respectively. The average age of MSEs was 3.6 years and the mean loan size of MSEs was 74438.18 Birr in the study area. The mean level of enterprise manager education was 10 years of schooling with a minimum of 6 and a maximum of 17 years of schooling.

The t-statistics result showed that there was a significant difference between non-defaulter and defaulters in enterprise manager education, distance to the financial institution, financial literacy, and loan supervision at 1% significance level. In addition, there was a significant difference between non-defaulter and defaulters in terms of collateral security, repayment period, loan utilization, and additional income source. Financial ratios results showed that the current ratio of enterprises is less than one (0.65) on average in the study area, which indicates MSEs have a liquidity problem in repaying their current liabilities from their current asset. The average debt ratio and debt-to-equity ratio for MSEs in the study area were 0.72 and 7.95, respectively. The higher ratio of debt financing raises the level of interest and principal repayment that increases the exposure of being defaulters. The logit regression estimate showed that manager education level, collateral security, and financial literacy positively and significantly affected loan repayment performance while distance to lending institutions, repayment period, and loan diversion negatively affected it.

## 5 Recommendation

Financial institutions should strongly consider loan and business characteristics that greatly influence loan repayment before they grant these loans to reduce the incidence of loan defaults. Therefore, the study recommends that:

➢ Financial institutions should provide orientation and training to enhance the financial management, savings and book-keeping skills of borrowers. In addition, these institutions should organize collateral security for their loans before loans are disbursed to reduce the risks of loan defaults.
➢ Lending institutions should regularly follow up and monitor the proper utilization of loans to ensure that they use the loans for the intended purpose. This can be done through checking business account statements regularly as well as physically visiting businesses. This supervision and monitoring should be inclusive for remote enterprises to increase loan repayment of MSEs far from lending institutions.
➢ The repayment period should be arranged in terms of enterprise production and marketing period to increase loan repayment performance.

## Declarations

### Author contribution statement

Ebrahim Endris: Conceived and designed the experiments; Performed the experiments; Analyzed and interpreted the data; Contributed reagents, materials, analysis tools or data; Wrote the paper.

### Funding statement

Ebrahim Endris was supported by Woldia University.

### Data availability statement

Data will be made available on request.

### Declaration of interest's statement

The authors declare no conflict of interest.

### Additional information

No additional information is available for this paper.

## References

[bib1] Abuye M., Shiferaw M. (2019). Determinants of loan repayment performance: evidence from in small and Medium enterprises: in case of Gurage zone: Ethiopia. Int. J. Sci. Res. Pub.(IJSRP).

[bib2] Afolabi J.A. (2017). Analysis of loan repayment among small scale farmers in oyo state, Nigeria. J. Soc. Sci..

[bib3] Agbeko D., Blok V., Omta S.W.F., Van der Velde G. (2017). The impact of training and monitoring on loan repayment of microfinance debtors in Ghana. J. Behav. Experim. Fin..

[bib4] Alemayehu M., Lemma M. (2014). Assessment of factors affecting the performance of microfinance institutions: the case of hawassa city. JBAS.

[bib5] Alemu O. (2018). Determinants of loan repayment of micro and small enterprises in jimma town, Ethiopia. Global J. Manag. Bus.: B Econom. Commer..

[bib6] Amene T.B. (2017). Factors affecting access to finance for micro and small enterprises: the case of West Hararghe Zone, Ethiopia. Int. J. Curr. Res..

[bib7] Amos S., Zanhouo D.A.K. (2019). Financial constraints, firm productivity and cross-country income differences: evidence from sub-Sahara Africa. Borsa Istanbul Rev..

[bib8] Baidoo S.T., Yusif H., Ayesu E.K., Mensi W. (2020). Improving loan repayment in Ghana: does financial literacy matter?. Cog. Econom. Fin..

[bib9] Baltagi B.H. (2011).

[bib10] Chirkos A.Y. (2014). The impact of microfinance on living standards, empowerment and poverty alleviation of the poor people in Ethiopia, A case study in ACSI. Res. J. Finance Account..

[bib11] Cochran W.G. (1977). United States of America.

[bib12] CSA (2007).

[bib13] CSA (2013).

[bib14] Dangisso M.Y., Deyganto K.O. (2020). Assessing the institutional outreach and sustainability of micro finance institutions in Ethiopia: evidence from omo microfinance institution hawassa branch. Am. J. Theor. Appl. Bus..

[bib15] Das P. (2019).

[bib16] Dougherty C. (2001).

[bib17] Enimu S., Eyo E.O., Ajah E.A. (2017). Determinants of loan repayment among agricultural microcredit finance group members in Delta state, Nigeria. Finan. Innov..

[bib18] FDRE (2016). Federal urban job creation and food security agency establishment council of ministers regulation. Feder. Democr. Repub. Ethiop..

[bib19] FDRE (2017).

[bib20] Ghosh R., Sen K.K., Riva F. (2020). Behavioral determinants of nonperforming loans in Bangladesh. Asi. J. Account. Res..

[bib21] Greene W.H. (2012).

[bib22] Gujarati D.N. (2004).

[bib23] Henock M.S. (2019). Financial sustainability and outreach performance of saving and credit cooperatives: the case of Eastern Ethiopia. Asia Pac. Manag. Rev..

[bib24] Inekwe J.N. (2019). Lending risk in MFIs: the extreme bounds of microeconomic and macroeconomic factors. J. Small Bus. Manag..

[bib25] Jote G.G. (2018). Determinants of loan repayment: the case of microfinance institutions in Gedeo zone, SNNPRS, Ethiopia. Univer. J. Account. Finan..

[bib26] Kebede M., Tegegn T., Tafese T. (2016). Factors affecting loan repayment performance of small scale enterprises financed by micro finance institutions: study on private borrowers around wolaita and Dawuro zone. Global J. Manag. Bus.: C Finance.

[bib27] Kim J.-H. (2015). A study on the effect of financial inclusion on the relationship between income inequality and economic growth. Emerg. Mark. Finance Trade.

[bib28] Lakew D.M. (2018). Financing practices of micro and small enterprises in west oromia region, Ethiopia. J. Manag. Res..

[bib29] Li H., Campbell D., Erdem S. (2022). Measuring time preferences using stated credit repayment choices. J. Quant. Econ..

[bib30] Mandefro G. (2010).

[bib31] Melese M., Asfaw M. (2019). Determinants of loan repayment performance of omo microfinance institution: in the case of mizan aman town, Southwest Ethiopia. Res. J. Bus. Manag..

[bib32] Mengstie B., Singh A. (2019). Impact of micro finance through Amhara credit and saving institution on women economic empowerment. Int. J. Recent Technol. Eng..

[bib33] Moritán M.G. (2020). Financial inclusion for MSMEs and women’s economic empowerment. J. Int. Counc. Small Busin..

[bib34] Nega F., Hussein E. (2016).

[bib35] NWZ-ASCI (2021).

[bib36] NWZEDO (2019).

[bib37] NWZEDO (2021).

[bib38] Ofori-Abebrese G., Baidoo S.T., Essiam E., McMillan D. (2020). Estimating the effects of financial inclusion on welfare in sub-Saharan Africa. Cog. Busin. Manag..

[bib39] Pasha A.M., Negese T. (2014). Performance of loan repayment determinants in Ethiopian micro finance - an analysis. Eurasi. J. Busin. Econom..

[bib40] Salifu A.T., Tofik-Abu Z., Rahman M.A., Sualihu M.A. (2018). Determinants of loan repayment performance of small and Medium enterprises (SMEs) in Ghana: the case of asante akyem rural bank. J. Afr. Bus..

[bib41] Sha'ban M., Girardone C., Sarkisyan A. (2019). Cross-country variation in financial inclusion: a global perspective. Eur. J. Finance.

[bib42] Shahriar S., Qian L., Rahman A., Hasan M., Kea S., Abdullahi N.M. (2019). Youth skill development loans (YSDL) and good governance in Bangladesh: a logit model analysis. Emerg. Mark. Finance Trade.

[bib43] Sissay F. (2016).

[bib44] Tegambwage A.G., Kasoga P.S. (2022). Loan repayment among group borrowers in Tanzania: the role of relationship quality. Future Busin. J..

[bib45] Ulwodi D.W., Muriu P.W. (2017). Barriers of financial inclusion in sub-Saharan Africa. J. Econ. Sustain. Dev..

[bib46] Wassie S.B., Kusakari H., Sumimoto M. (2019). Performance of microfinance institutions in Ethiopia: integrating financial and social metrics. Soc. Sci..

[bib47] WB (2020).

[bib48] Yuanyuan H., Zongxian F. (2019). Is collective financing feasible for small and micro-sized enterprises? An evolutionary game analysis of the credit market in China. Econom. Res. Ekonomska Istraživanja.

